# Repeated dose multi-drug testing using a microfluidic chip-based coculture of human liver and kidney proximal tubules equivalents

**DOI:** 10.1038/s41598-020-65817-0

**Published:** 2020-06-01

**Authors:** Ni Lin, Xiaobing Zhou, Xingchao Geng, Christopher Drewell, Juliane Hübner, Zuogang Li, Yingli Zhang, Ming Xue, Uwe Marx, Bo Li

**Affiliations:** 1 Key Laboratory of Beijing for Safety Evaluation of Drugs, National Center for Safety Evaluation of Drugs, National Institutes for Food and Drug Control, A8 Hongda Middle Street, Beijing Economic-Technological Development Area, Beijing, 100176 P. R. China; 2 Department of Pharmacology, Beijing Laboratory for Biomedical Detection Technology and Instrument, School of Basic Medical Sciences, Capital Medical University, Beijing, 100069 China; 3 Technische Universitaet Berlin, Institute of Biotechnology, Department Medical Biotechnology, Gustav-Meyer-Allee 25, 13355 Berlin, Germany; 4 TissUse GmbH, Oudenarder Strasse 16, 13347 Berlin, Germany; 5 National Institutes for Food and Drug Control, 31 Hua Tuo road, Daxing district, Beijing, 102629 China; 6 Beijing Institute for Drug Control, 25 Science Park Road, Changping District, Beijing, 102206 China

**Keywords:** Tissue engineering, Biomarkers, Pharmacology, Toxicology

## Abstract

A microfluidic multi-organ chip emulates the tissue culture microenvironment, enables interconnection of organ equivalents and overcomes interspecies differences, making this technology a promising and powerful tool for preclinical drug screening. In this study, we established a microfluidic chip-based model that enabled non-contact cocultivation of liver spheroids and renal proximal tubule barriers in a connecting media circuit over 16 days. Meanwhile, a 14-day repeated-dose systemic administration of cyclosporine A (CsA) alone or in combination with rifampicin was performed. Toxicity profiles of the two different doses of CsA on different target organs could be discriminated and that concomitant treatment with rifampicin from day6 onwards decreased the CsA concentration and attenuated the toxicity compared with that after treatment with CsA for 14 consecutive days. The latter is manifested with the changes in cytotoxicity, cell viability and apoptosis, gene expression of metabolic enzymes and transporters, and noninvasive toxicity biomarkers. The on chip coculture of the liver and the proximal tubulus equivalents showed its potential as an effective and translational tool for repeated dose multi-drug toxicity screening in the preclinical stage of drug development.

## Introduction

Drug-induced nephrotoxicity and hepatotoxicity are associated with substantial morbidity and mortality and is a common reason for drug withdrawal^1,2^. The kidney plays a critical role in sustaining the electrolyte balance and addressing filtration, secretion and reabsorption. The renal proximal tubule is responsible for the clearance of xenobiotics via vectorial transport (reabsorption or secretion), owing to the strong polarization of the proximal tubule epithelial cells expressing functional transporters^3^. As another vital organ, the liver controls the metabolism of xenobiotics; while the kidney is responsible for their elimination. The plasma concentration and the bioavailability of xenobiotics are altered via hepatic toxification/detoxification, resulting in a change in nephrotoxicity. Therefore, the renal proximal tubule and liver represent major targets for toxic compounds. Current preclinical models for demonstration of drug nephrotoxicity and hepatotoxicity involve *in vitro* and *in vivo* models. However, Animal models show limitations due to phylogenetic distance and animal ethics. The conventional methods of cell culture in static conditions are unable to mimic the *in vivo*-like microenvironment^4^.

In support of the ToxCast/Tox21 Initiative Strategy^5^, more predictable toxicity assessment methods in a high throughput manner need to be explored. The development of human organ chip technology overcomes the drawbacks of current preclinical models and supports the 3R principles (that is, replacement, reduction and refinement of animals)^6,7^. The present human multi-organ-chip is integrated with peristaltic pumps and media containers, leading to a fluid-to-tissue ratio similar to that in humans^8^. The liver-proximal tubule coculture chip allows the distribution and exchange of nutrients, compounds and their metabolites; thus, the communication between the two tissue equivalents is guaranteed. The homeostatic steady-state ensures that the observation of different stages of liver and/or kidney damage can be achieved. Additionally, the utilized differentiated human renal proximal tubule cells can form an integrated barrier and express functional enzymes and transporters essential to drug-induced kidney injuries and drug interactions^9,10^. The coculture liver spheroids were shown to express liver-specific markers, human enzymes and transporters at levels comparable to or even higher than those of 2D culture cells for at least 28 days^11^.

Cyclosporine A (CsA), an immunosuppressant with nephrotoxic and hepatotoxic effect^12,13^, was used as a model drug to challenge the chip-based liver-proximal tubule coculture system. A repeated treatment with a combination of CsA and rifampicin (RFP), a well-known inducer of hepatic enzymes and transporters^14^, was applied to further evaluate drug metabolism and toxicity on the two-organ-chip (2-OC). The results of studies on the pharmacology, pharmacokinetics and toxicity of CsA showed major variations between species, especially in the metabolic profiles^15–18^. Therefore, two different CsA dosages (nontoxic/toxic dosage) and a therapeutic dosage of RFP were applied to the coculture chip model, according to the dosage used in clinical trials^10,12,19,20^.

To the best of our knowledge, this is the first study to show that a microfluidic chip-based liver-proximal tubule coculture model can be used to perform a toxicity study with repeated administration alone or in combination for 14 consecutive days. The present liver-proximal tubule coculture chip could effectively mimic the process of drug interaction and its influence on toxicity in the human body. The results indicated that the liver-proximal tubule coculture chip shows promise in elucidating the drug interaction, metabolism and toxicity in combination with detecting the morphology, histopathology, molecular biology, drug metabolism, and noninvasive toxicity biomarkers.

## Results

**Figure 1:**
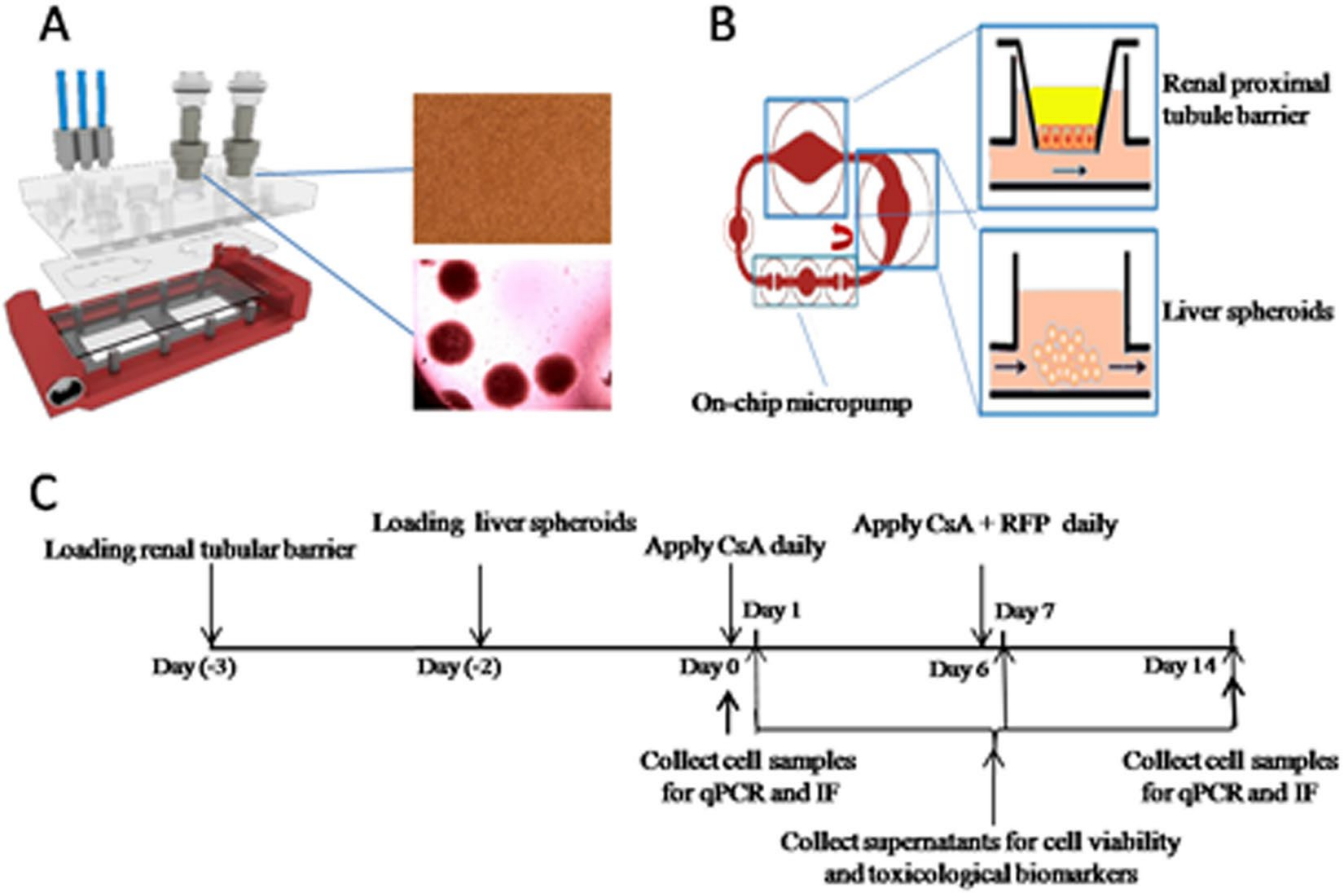
The microfluidic live-proximal tubule two-organ-chip. (**A**) Expanded view of the device comprising the PDMS-glass chip accommodating two microfluidic circuits. (**B**) Direction of fluid flow within the circuit is illustrated by red bold arrows and the experimental set up of cocultivation of liver microtissues and renal proximal tubule barriers in the MOC. (**C**) Schematic overview of the 16-day coculture, including a 2-day adaptation period and a 14-day repeat application of compounds, with daily media exchange, supernatant collection on days 1, 7 and 14, and cell sample collection on days 0 and 14.

We designed a microfluidic 2-OC accommodating two identical circuits, each interconnecting a kidney proximal tubule barrier insert with a 40-spheriods liver equivalent for subsequent coculture (Fig. 1A-B).

### Characterization, integrity and function of renal proximal tubule barriers

**Figure 2:**
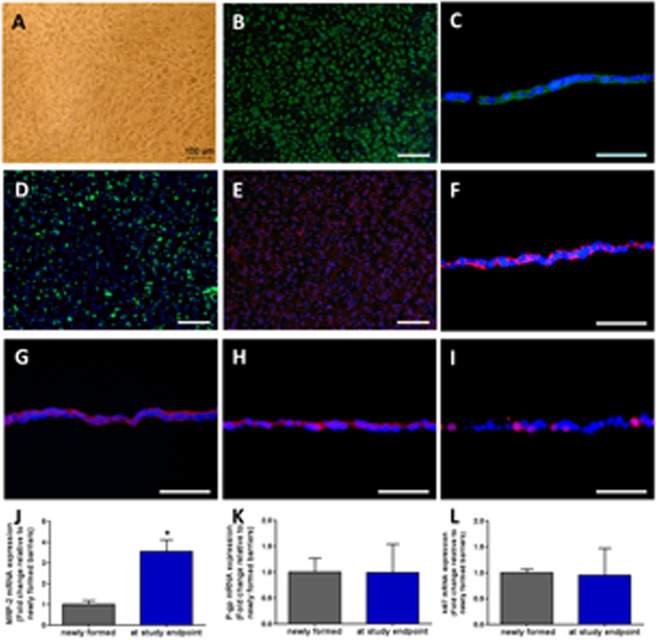
Characterization of proximal tubule barriers. (**A**) The formation of a proximal tubule monolayer on a permeable support insert. (**B**) CK8/18 (green) (**C**) CK8/18 (green), cross section (**D**) ace-tubulin (green) (**E**) MRP-2 (red) (**F**) Na + -K + -ATPase (red) (**G**) ZO-1 (red) (**H**) Claudin-10 (red) (**I**) Ki67 (red). Nuclei were stained with DAPI (blue). Scale bars B, D, and E: 100 μm; C, F, G, H and I: 50 μm. qRT-PCR analysis of (**J**) MRP-2 (**K**) P-gp (**L**) Ki67 gene expression. The fold changes in mRNA expression of the barriers at the endpoint are represented relative to barriers newly formed; data are the mean ± SEM, experiments were performed in triplicates.

The RPTEC/TERT1 cells reached confluence on membrane inserts to achieve differentiation and form an integral monolayer (Fig. 2A), as described in previous protocols^11^. When the integral barrier newly formed, immunofluorescence staining was performed. RPTEC/TERT1 cells displayed clear staining of the pan-epithelial marker CK8/18 (Fig. 2B,C). Formation of primary cilia was observed by staining for ace-tubulin (Fig. 2D). Figure 2E and Fig. 2F demonstrated the localization of MRP-2 and Na^+^-K^+^-ATPase, respectively, which are indicators of a functionally polarized RPTEC monolayer. The expression of ZO-1 (Fig. 2G) and Claudin-10 (Fig. 2H) at the cell borders confirmed the tight junction formation between neighboring RPTEC/TERT1 cells and indicated appropriate cell polarization as well as the integrity of the barrier. Positive staining for Ki67 (Fig. 2I) showed the proliferating cells.

RT-qPCR analysis of functional transporters MRP-2, P-gp and Ki67 were illustrated in Fig. 2J-L, as control of changes in gene expression during a 16-day cultivation in 2-OCs.

### Characterization and function of liver spheroids

Morphology and differentiation of HepaRG cells, formation of liver spheroids, immunofluorescence staining and RT-qPCR analysis of functional hepatic enzymes and transporters were shown in Supple. Figure 1. The results showed a long-term maintenance and reproducible differentiation characteristic and suggested a balance between cell proliferation, differentiation and apoptosis, leading to a physiological homeostasis of liver spheroids in the microfluidic multi organ chip.

### Performance of cocultures exposed to fluid flow over sixteen days

**Figure 3:**
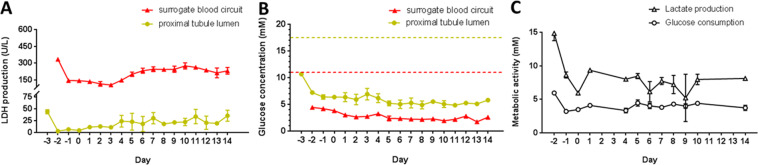
Performance of the fluid flow-exposed liver-proximal tubule coculture system over 16 days. (**A**) Systemic tissue viability in the 2-OC represented by LDH production in the surrogate blood circuit (red) and the proximal tubule lumen (yellow). (**B**) Glucose concentration balance in the surrogate blood circuit (red) and the proximal tubule lumen (yellow) of the 2-OC. Dotted lines represent the respective initial glucose concentration of the media added daily into the liver compartment (red) and proximal tubule lumen (yellow) (**C**) Metabolic activity of cocultivation measured by lactate production and glucose consumption in the circulating media.

The LDH production in the proximal tubule lumens and in the surrogate blood circuits were evaluated separately (Fig. 3A). LDH production levels in the 2-OC normalized toward a constant steady state in the excretory lumen and the surrogate blood circuit, indicating an artificial but stable physiological cell turnover within the different organ compartments over the whole cocultivation period of 16 days.

Glucose levels measured in the two media pools over 16 days of coculture are summarized in Fig. 3B. The glucose concentrations in the blood circuit and excretory lumen were narrowly maintained between 2.5 mM and 6 mM to ensure that an adequate amount of energy was provided to the equivalents of the 2-OC system. The glucose level in the proximal tubule lumen was decreased approximately threefold compared to the initial medium content of 17.5 mM. The glucose concentration in the blood circuit constantly decreased to 50% of the initial 11.1 mM glucose, which was within the narrow range of normal blood glucose. These findings demonstrated that a remarkably stable glucose balance was established in the 2-OC system throughout the 16-day coculture of liver and proximal tubule equivalents.

Glucose consumption and lactate production in the surrogate blood circuit were measured as indicators of metabolic activity of the coculture system (Fig. 3C). The metabolic activity of the 2-OC system showed a biphasic profile with a period of constant decrease in the first two administration-free days and a steady state with minor fluctuations from day zero onwards. The slight decrease in lactate production in the surrogate blood circuit might indicate conditional effects between the liver spheroids and proximal tubule barrier.

Both the cell viability and metabolic profile proved that the 2-OC system can cultivate a combination of the two organ equivalents successively for 16 days while maintaining their viability and characteristic functions. The establishment of a microfluidically linked coculture system in a reproducible and robust manner facilitates the application of substances in subsequent experiments.

### Modeling systemic fourteen-day repeated-dose substances exposure to the chip-based liver-renal proximal equivalents cocultivation

#### Toxicity profile of test substances in liver-proximal tubule cocultures in the 2-OC

**Figure 4:**
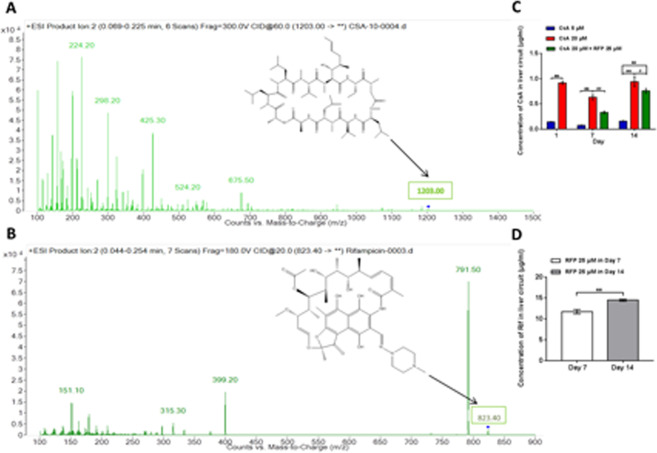
Measurement of the concentration of CsA and RFP after daily administration or coadministration and metabolism by liver-proximal tubule cocultured equivalents in 2-OC. (**A**) Mass spectrum of CsA in circulating media. The characteristic peak of CsA is represented by an arrow. (**B**) Mass spectrum of RFP in circulating media. The characteristic peak of RFP is represented by an arrow. (C) Concentration of CsA in the circulating media after repeated administration/coadministration daily and metabolization by liver-proximal tubule cocultured equivalents in the 2-OC on days 1, 7 and 14. (**D**) Concentration of RFP in the circulating media after repeated daily coadministration and metabolism by liver-proximal tubule cocultured equivalents in 2-OC on days 7 and 14. Data are the mean ± SEM of three (control and low dose groups) or four (high dose and coadministration groups) biological replicates. * (#) or ** (##) indicates significant difference p < 0.05 or p < 0.01, respectively. * or #indicates differences compared with the corresponding controls or when the coadministration group was compared with the high-dose group, respectively.

Chip-based cocultures of liver and renal proximal tubule equivalents were treated with CsA or CsA combined with RFP to observe tissue interactions during varying levels of toxic stress. Samples were taken for detection of the concentration of CsA (Fig. 4A) or RFP (Fig. 4B) post-metabolism by liver spheroids at 24 hours after the first (day1) and the last (day14) day of administration and after the first day of coadministration (day7). As illustrated in Fig. 4C, exposure of liver equivalents to CsA in the surrogate blood circuit increased as the dose level increased from 5 to 20 μM at 24 hours after administration on days 1, 7 and14. The CsA level at 24 hours after consecutive administration for seven days was lower than that after the first and the last administration. The concentration of CsA in the surrogate blood circuit with administration of 20 μM CsA decreased when it was applied in systemic combination with 25 μM RFP after the first and the last coadministration. After concomitant administration of RFP for 8 days, the reduction in the CsA concentration between circuits with or without RFP was attenuated. Meanwhile, the concentration of RFP increased after a daily coadministration for 8 days (Fig. 4D), indicating accumulation of RFP in the surrogate blood circuit.

**Figure 5:**
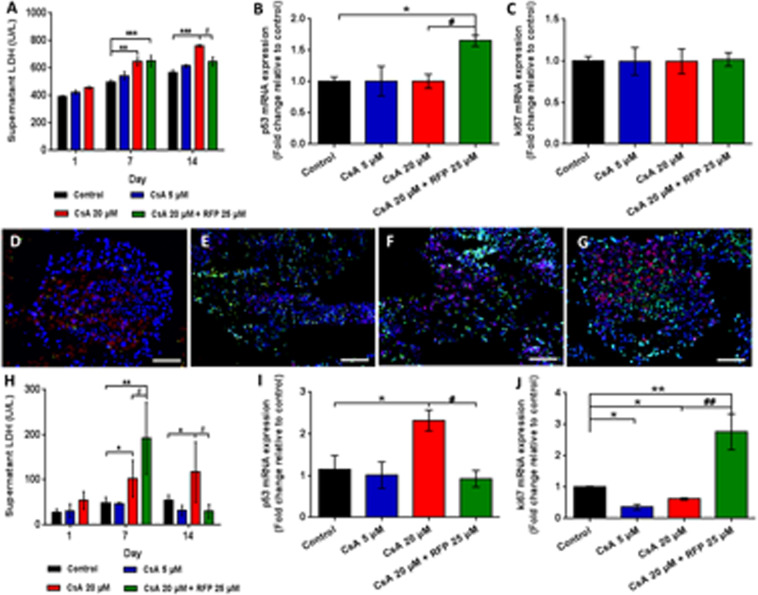
Toxicity profile of daily administration of CsA alone or with concomitant use of RFP in liver-proximal tubule coculture chips for 14 days. (**A**) LDH activity in surrogate blood media on days 1, 7 and 14. qRT-PCR analysis of (**B**) p53 (C) Ki67 gene expression in liver spheroids at the endpoint of the experiment. TUNEL/Ki67 (green/red) staining of liver spheroids in (**D**) control, (**E**) low-dose (**F**) high-dose and (**G**) coadministration groups at the endpoint of the experiment. (H) LDH activity in excretory media on days 1, 7 and 14. Nuclei were stained with DAPI (blue).Scale bars D and G: 100 μm, E and F: 200 μm. qRT-PCR analysis of (I) p53 and (**J**) Ki67 gene expression in proximal tubule barriers at the endpoint of the experiment. Data are the mean ± SEM, experiments were performed in triplicates (control and low-dose groups) or quadruplicate (high-dose and coadministration groups). * (#) or ** (##) indicates significant difference p < 0.05 or p < 0.01, respectively. * or #indicates differences compared with the corresponding controlsor when the coadministration group was compared with the high-dose group, respectively.

To assess the effect of model substances on liver spheroids and on the RPTEC/TERT1 cell monolayer following liver metabolism, we examined LDH release, as a reliable biomarker for the assessment of nephrotoxicity *in vitro*, in the two media pools. The LDH concentration in the surrogate blood circuit increased gradually as the CsA concentration increased on days 1, 7 and 14 (Fig. 5A). The LDH levels in circuits administered systemically with 20 μM CsA decreased after coadministration of 25 μM RFP for 8 consecutive days, consistent with the concentrations of CsA in surrogate blood circuits; however, this decrease was not observed after the first coadministration (Fig. 5A). At the endpoint of the experiment, the p53 gene expression in the coadministration group showed a 1.5-fold increase compared to that in the untreated controls, while no change was observed in the expression of Ki67 (Fig. 5B,C). However, the LDH release in the excretory pool revealed dramatic but not significant increases (Fig. 5H). After the first coadministration, the LDH level continued to increase compared with that in untreated controls on day7 (Fig. 5H). However, a dramatic decline in LDH level could be observed after the concomitant use of RFP for 8 days (Fig. 5H). Furthermore, the expression of the p53 gene in RPTEC/TERT1 cells treated with a high dose of CsA for 14 days showed a 2.3-fold increase compared with that of control cells (Fig. 5I,J). A 2.7-fold increase in the expression of the Ki67 gene in renal cells treated concomitantly with CsA and RFP was observed, while significant decreases in Ki67 expression were found in both the low- and high-dose groups at the experimental endpoint (Fig. 5I,J). As illustrated in Fig. 5D–G, TUNEL/Ki67/DAPI triple staining revealed increased cell death by CsA exposure, while an alleviation of cell death could be observed in liver spheroids administered CsA in combination with RFP. Proliferating cells visualized by Ki67-positive staining were evenly distributed in liver spheroids and maintained in the chips and showed no significant change between the different treatment groups (Fig. 5D–G).

#### Protein expression of conventional and novel noninvasive biomarkers of toxicity

To explore the further application of the MOC platform for the early detection and diagnosis of organ-specific toxicity, we examined conventional and novel biomarkers that are approved for clinical practice to determine the drug-induced liver and kidney injury in the present experiment. Observations of the changes in levels of biomarkers were tabulated in Supple. Tab. 3.

##### Biomarkers in the surrogate blood

**Figure 6:**
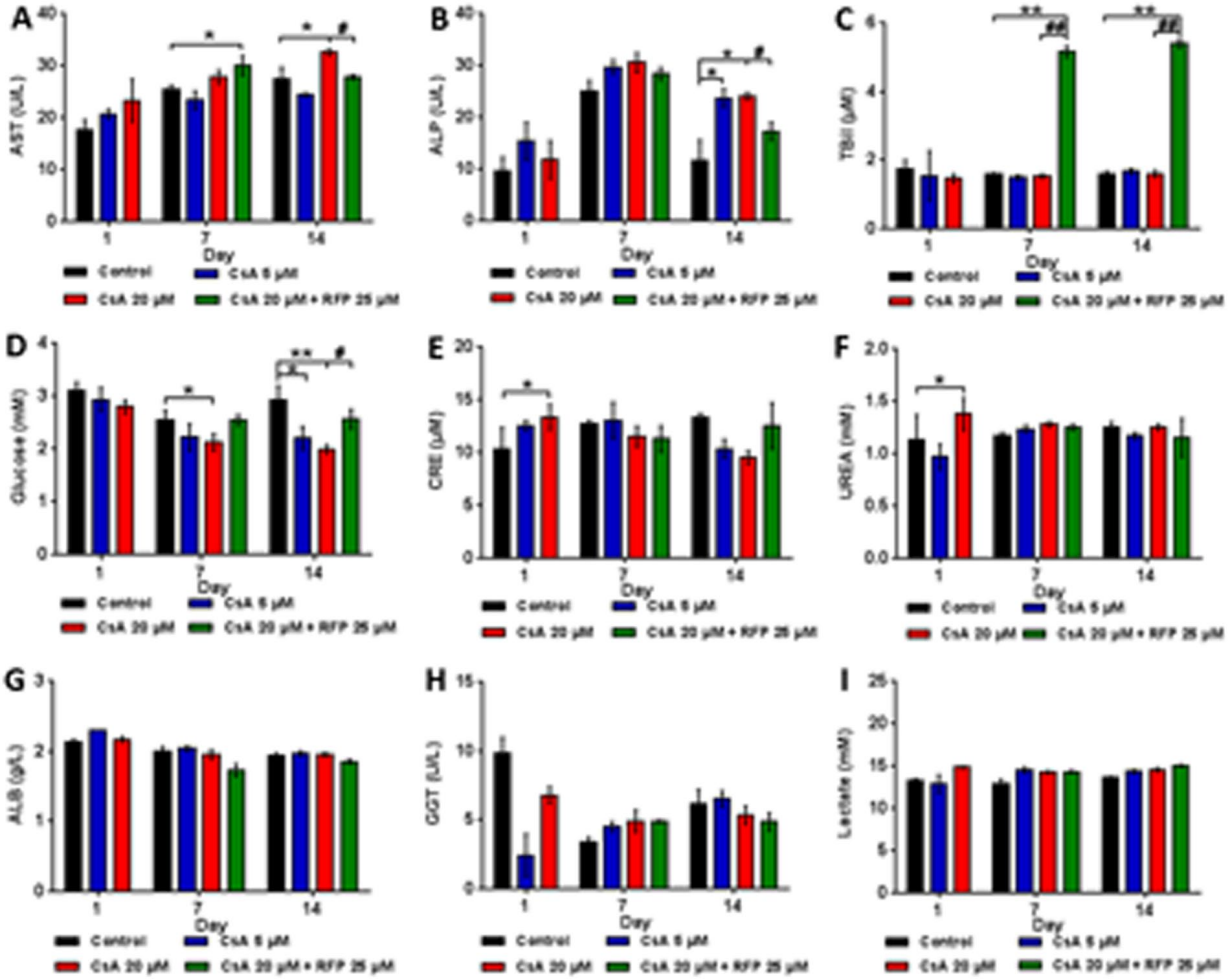
Protein expression of noninvasive toxicity biomarkers in surrogate blood after daily administration of CsA alone or with concomitant use of RFP in liver-proximal tubule coculture chips for 14 days. Protein level of (**A**) AST (**B**) ALP (**C**) TBiL (**D**) Glucose (**E**) CRE (**F**) UREA (**G**) ALB (**H**) GGT (**I**) Lactate in circulating media on days 1, 7 and 14. Data are the mean ± SEM, experiments were performed in triplicates (control and low-dose groups) or quadruplicate (high-dose and coadministration groups). * (#) or ** (##) Indicates significant difference p < 0.05 or p < 0.01, respectively. * or # Indicates differences compared with the corresponding controls or when the coadministration group was compared with the high-dose group, respectively.

In the surrogate blood media (Fig. 6), the hepatic enzyme AST dose-dependently increased, and the AST level in the coadministration group revealed a uction at the endpoint rather than after the first dose of RFP. ALP increased after treatment with CsA and showed a significant change only on day14, while concomitant treatment with RFP reduced the ALP level when administered with 20 μM CsA. Dramatic increases in TBiL could only be observed during the post-RFP stage. The glucose in surrogate blood showed dose-dependent decreases, and these molecules reached statistically significant levels in the high-dose group on day7 and in both of the treatment groups on day14. After concomitant treatment with 20 μM CsA and RFP, the concentration of glucose in the surrogate blood circuit increased during the coadministration stage. The nephrotoxicity biomarkers CRE and UREA in surrogate blood increased on day1; however, no drug-related changes were observed on days 7 and 14. No CsA-related changes were detected in the levels of ALB, GGT and Lac.

##### Biomarkers in the excretory pool

**Figure 7:**
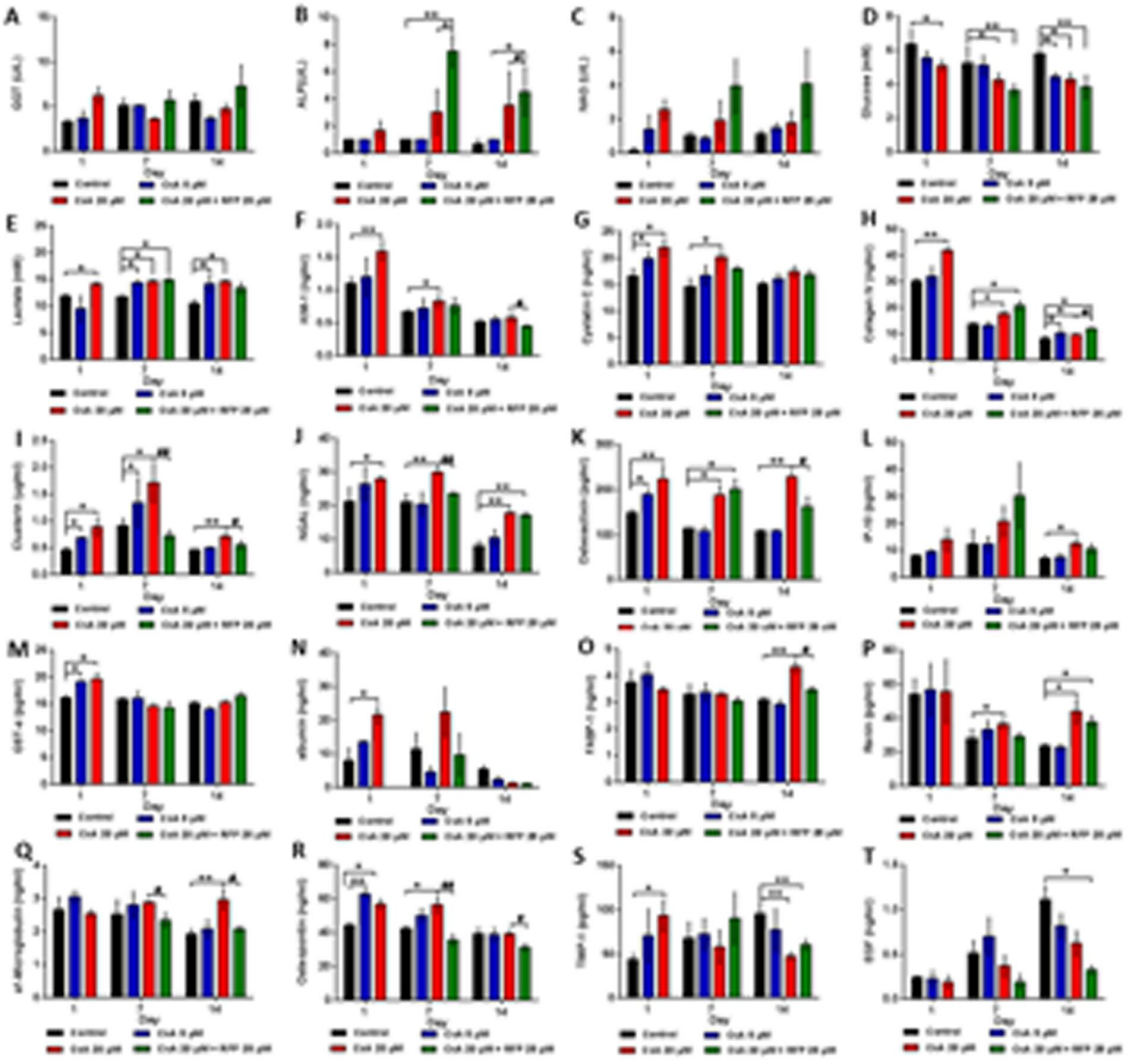
Protein expression of noninvasive toxicity biomarkers in the excretory lumen after daily administration of CsA alone or with concomitant use of RFP in liver-proximal tubule coculture chips for 14 days. Protein levels of (**A**) GGT (B) ALP (**C**) NAG (**D**) Glucose (**E**) Lactate (**F**) KIM-1 (**G**) Cystatin C (H) Collagen IV (**I**) Clusterin (**J**) NGAL (**K**) Osteoactivin (**L**) IP-10 (**M**) GST-α (**N**) albumin (**O**) FABP-1 (**P**) Renin (**Q**) α1-microglobulin (**R**) Osteopontin (**S**) TIMP-1 (**T**) EGF in excretory media on days 1, 7 and 14. Data are the mean ± SEM, experiments were performed in triplicates (control and low-dose groups) or quadruplicate (high-dose and coadministration groups). * (#) or ** (##) indicates significant difference p < 0.05 or p < 0.01, respectively. * or # indicates differences compared with the corresponding controls or when the coadministration group was compared with the high-dose group, respectively.

In the excretory media (Fig. 7), the GGT levels showed a dose-dependent increase on day1. However, after seven successive treatments of CsA, the GGT level of the high-dose group obviously decreased, and a similar effect was also observed on day14. Concomitant treatment with RFP did not result in a reduction in GGT levels on days 7 and 14. Part of the proximal tubule barriers in all of the treatment groups secreted the urinary enzyme NAG as the CsA dose increased. Similar effects of CsA and RFP were found on ALP in the excretory pool. Glucose dose-dependently decreased under the effect of CsA, and the glucose level in the coadministration group was lower than that in the CsA treatment groups. The concentration of Lac significantly increased after treatment with CsA, while RFP alleviated the effect of CsA on Lac secretion.

With respect to potential novel urinary biomarkers, KIM-1, the proximal tubule-specific biomarker that has been formally qualified by regulatory agencies^21^, was dose-dependently increased throughout the study period, and the CsA-mediated increase in KIM-1 abated with time, as well as with concomitant administration of RFP. Similar results could also be observed on the CysC and Col IV levels in excretory media; however, RFP enhanced Col IV during the post-RFP stage. The concentrations of Clu and NGAL were significantly increased after treatment with CsA, and RFP obviously decreased the Clu and NGAL levels. In addition, the levels of these two biomarkers reached the highest level of secretion and the strongest reduction by using RFP in the middle stage of the study. The secretion level of OA was sustainable during the experimental period, and the OA level in the high-dose group was maintained at a high level. The reduction in OA in the coadministration group showed delayed significance. Similarly, IP-10 was increased after treatment with 20 μM CsA, while RFP slightly decreased IP-10 on day14. The dose-dependent increases induced by CsA in GST-α and albumin levels could only be observed on day1. CsA enhanced FABP-1, and the reduction produced by concomitant treatment with RFP was not observed until the late stage (day14), while similar drug-induced changes in REN and α1-MG occurred on days 7 and 14. Administration of CsA caused a dose-independent increase in OPN on day1, while on day7, OPN increased in a dose-dependent manner. The significant reductions in OPN induced by RFP were maintained during the post-RFP stage, although the effect of CsA on OPN disappeared on day14. TIMP-1 showed a dose-dependent significant increase on day1; however, the effect of CsA on TIMP-1 was reversed on days 7 and day14. In the post-RFP stage, the increase caused by CsA in the TIMP-1 level was weakened to different degrees. EGF dose-dependently decreased after treatment with CsA or concomitant treatment with RFP. No drug-related changes were observed in CALB and TFF-3 (data not shown).

#### Changes in gene expression of metabolic enzymes and transporters

**Figure 8:**
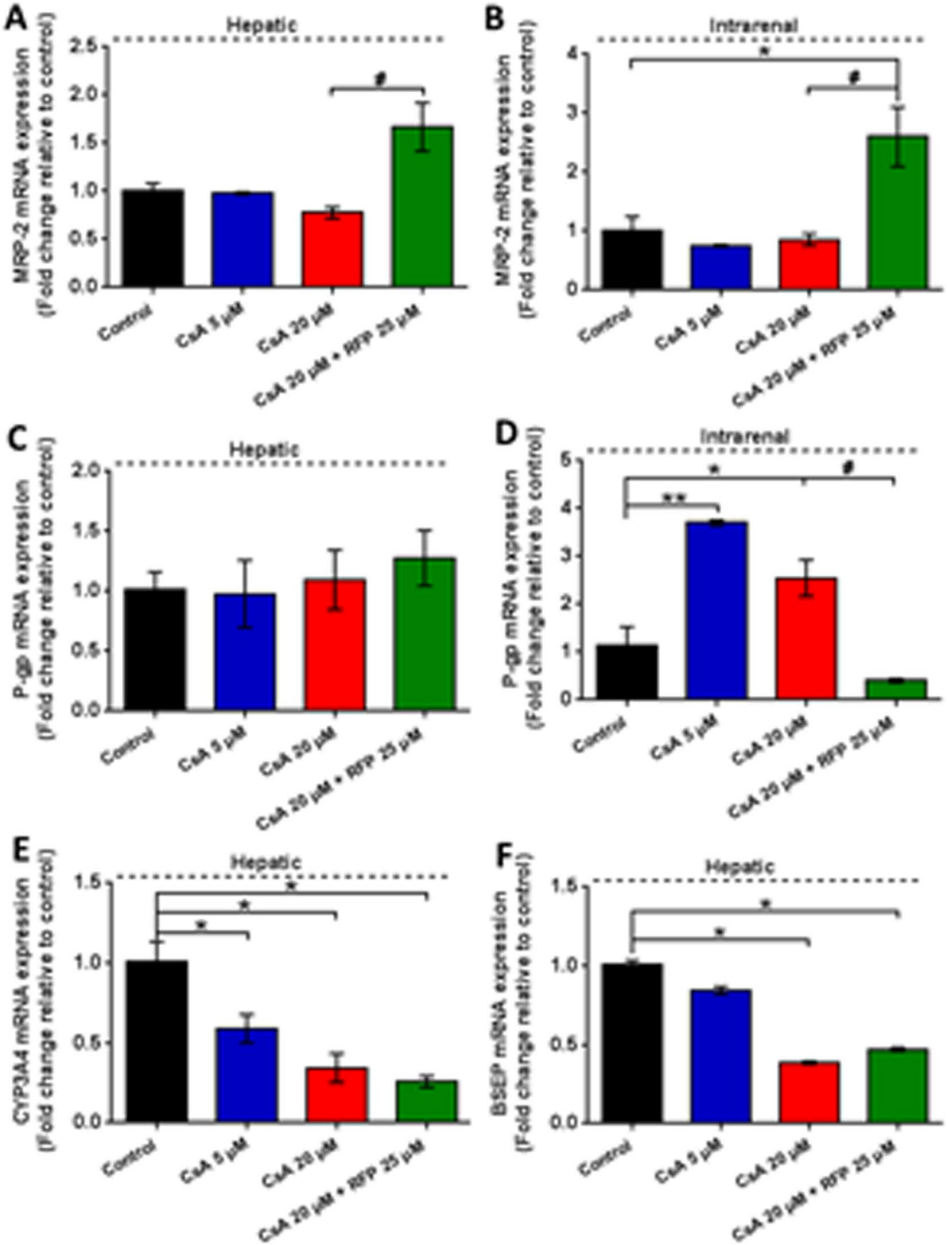
Gene expression of metabolic enzymes and transportersin liver spheroids and proximal tubule barriers after daily administration of CsA alone or with concomitant use of RFP in liver-proximal tubule cocultures chips for 14 days. The fold changes in mRNA expression of hepatic (**A**) MRP-2 (**C**) P-gp (E) CYP3A4 (F) BSEP and intrarenal (**B**) MRP-2 (**D**) P-gp, normalized to the control group, at the endpoint of the experiment. Data are the mean ± SEM, experiments were performed in triplicates (control and low-dose groups) or quadruplicate (high-dose and coadministration groups). * (^#^) or ** (^##^) indicates significant difference *p* < 0.05 or *p* < 0.01, res*p*ectively. * or ^#^indicates differences compared with the corresponding controls or when the coadministration group was compared with the high-dose group, respectively.

We further investigated the functional enzymes and transporters expressed in two equivalents and their roles in detoxification or toxification processes in the present study (Fig. 8). At the end of the experiment, concomitant treatment with RFP significantly induced the expression of MRP-2 in liver spheroids and proximal tubule barriers. P-gp gene expression in liver spheroids was slightly increased after coadministration with RFP. In contrast, the expression of the P-gp gene in RPTEC/TERT1 cells could be dramatically induced by successive treatment with CsA, while in the coadministration group, the induction of the P-gp gene could not be observed. Statistically significant and dose-dependent decreases in the expression of the CYP3A4 and BSEP were observed in the CsA-treated groups. The inhibitory effect of CsA on BSEP was slightly attenuated by the coadministration of RFP, while no significant difference was found in CYP3A4 between the CsA group and the concomitant treatment group.

## Discussion

Several recent studies have demonstrated that organ-on-a-chip technology reconstructs a 3-dimensional (3D) microenvironment that can mimic the structural, mechanical, physiological and metabolic properties of the human organs and tissues^22–25^. For instance, liver tissue models established using 3D technology showed enhanced hepatic functions, especially when non-parenchymal cells were integrated^26–30^. We have developed techniques to aggregate stable and reproducible liver spheroids, consisting of differentiated HepaRG (parenchymal) and HHSteC (non-parenchymal) cells, and to reproduce hepatic lobule equivalents, that is, the functional units of the liver^24,31–34^. The HepaRG cell line was utilized as an *in vitro* model to predict cytochrome P450 (P450) enzyme induction of drugs in humans^35–37^. Furthermore, emulation of the fluid shear stress and interstitial pressure surrounding liver spheroids contributes to maintaining stable protein and oxygen gradient-based microenvironments for a prolonged period in chips^38–40^. Moreover, the expression of tight junction-related proteins, including ZO-1 and Claudin-10, indicated tight junction assembly and cell polarity of the proximal tubule cell barriers throughout the entire coculture.

For verification of the functional integrity of the proximal tubule cell barriers throughout the entire coculture, the glucose balance between the apical and the basolateral sides of the barriers with different concentrations of glucose challenges on both sides is a reliable indicator. We supplemented approximately 0.8 mg glucose in total per day to the two media pools, 0.5 mg and 0.3 mg into the liver compartment and the proximal tubule lumen, respectively, to evaluate the response of the equivalents at non-physiologically high levels of glucose. Furthermore, the production of lactate and consumption of glucose remained relatively steady in the coculture system during the administration period, demonstrating a successful establishment of an artificial but stable coculture of the two organ equivalents. Moreover, distinct LDH concentration gradients between the two media pools could be detected. We could hypothesize that LDH is not able to pass through an integral and functional proximal tubule cell monolayer due to its size (135–140 kDa)^41^. Consequently, as long as the barrier has integrity and is functional, LDH values on each side of the membrane should be exclusively generated by the physiological turnover of respective organ model. In addition, static controls of the liver-proximal tubule chip showed a disintegration of liver aggregates, damage of proximal tubule barriers, and dramatically increases in LDH concentrations of both two media pools from day6 or day7 (data not shown). Robust performance of the microfluidic-based liver-proximal tubule chip up to 16 days indicates this system can be used to screen the potential toxicity of xenobiotics.

We chose CsA as a model drug with concomitant systemic administration of RFP to evaluate drug metabolism and toxicity by the liver-proximal tubule chip. CsA has been identified as a substrate as well as an inhibitor of CYP3A4 and P-gp, which undergoes hepatic and intestinal metabolism in humans^42,43^, while the renal elimination of CsA mainly depends on intrarenal P-gp^12^. Many studies on CsA toxicity have been performed in animals^12,15^ and *in vitro*^16–18^ demonstrating interspecies differences that include P-gp mRNA expression in major metabolic organs^44,45^, BSEP mRNA expression in liver^46^ and the overestimation of human biliary excretion and clearance in animal model^47^. Additionally, chronic nephrotoxicity of CsA did not occur until repeated CsA treatments for prolonged periods in the clinic^12^. Therefore, CsA is suitable as a model substance for the liver-kidney-on-chip platform. RFP as an inducer of CYP3A4 and P-gp, could accelerate the metabolism of CsA resulting in attenuated toxicity to target organs, such as the liver and renal proximal tubule^14^.

In several clinical trials, a daily dosage of 10–15 mg/kg revealed several cases of nephrotoxicity and one case of hepatotoxicity^19,20^, while nephrotoxic side effects were not observed at low doses (2.5–5 mg/kg, daily intravenous) of CsA therapy^48,49^. The bioavailability decreased to 10% after concomitant use of RFP (600 mg/day) for 11 days^14,20^. The selection of the dosage of CsA applied in the coculture experiments was guided by following general assumptions: the cell numbers of liver spheroids and proximal tubule barriers represent approximately 1/100,000 of the size of the respective counterpart organ in the human body. Thus, the dosage in the MOC system corresponds to 20.8–31.2 μM/day (toxic doses of CsA), 5.2–10.4 μM/day (nontoxic doses of CsA), and 24.3 μM/day (nontoxic dose of RFP). In addition, considering the results of the preliminary coculture experiment in static conditions (data not shown) and several previous *in vitro* studies on hepatotoxicity and nephrotoxicity of CsA, 5 and 20 μM of CsA were used for the low- and high-dose groups, and 25 μM of RFP was used for the concomitant treatment group.

The major results of the exposure experiments were that the toxicity profiles of two different CsA doses on different target organs could be discriminated and that the coculture model could effectively mimic the process of drug metabolism and its influence on toxicity in the human body. Cocultures exposed to high concentrations of CsA showed a noticeable increase in LDH in excretory media, indicating the increase in the epithelial barrier permeability induced by CsA-induced injury. The increase in p53 gene expression in renal cells of the high-dose group revealed an induction in response to cellular stress caused by a toxic dose of CsA^50,51^. Interestingly, however, this phenomenon disappeared after coadministration of RFP for 8 consecutive days. We hypothesize that the decreased dose of CsA in the concomitant treatment with RFP reduced the toxicity and induced the proliferation of RPTEC/TERT1 cells and the reassembly of tightly packed renal tubular epithelial barriers. This hypothesis is supported by the results that the Ki67 mRNA expression in renal cells was significantly induced after repeated coadministration of CsA and RFP. A slight induction of the p53 gene in the liver spheroids of the coadministration group was speculated to be a signal of repair after toxic injury^52^, while no change in p53 mRNA expression was detected in CsA-treated liver spheroids after a 14-day repeated treatment. Similar to previous *in vitro* studies^53^, the alteration of the p53 gene depends on the dosage and the period of CsA treatment.

Furthermore, we investigated the expression of functional enzymes and transporters and their roles in detoxification or toxification processes. RFP is a well-known strong inducer of CYP3A4, which has been proven in studies *in vitro* and *in vivo*^54,55^; however, the induction of CYP3A4 by RFP was not observed. This result may be due in part to the induction effect of RFP, which during the post-RFP stage failed to reverse the inhibitory effect of high-dose CsA; however, a remarkable decrease in CsA concentration in surrogate blood and in the cytotoxic indicators LDH and ALP could be observed in the RFP coadministration group on day14. Similarly, the effects on hepatic P-gp by RFP and CsA were not significant at the endpoint. Interestingly, however, the effects on renal P-gp by the two model substances showed differences from those of hepatic P-gp. Renal P-gp expression plays a key role in the renal elimination of xenobiotics and shows a large interspecies variation^45^. However, the inductive or inhibitory effect of CsA on P-gp has long been controversial since several studies conducted on different animal and *in vitro* models drew different conclusions^56,57^. The results from the organ chip model suggested that CsA induces intrarenal P-gp, while the inductive effect decreased as the CsA dose increased. The slight inhibitory effect on intrarenal P-gp with the coadministration of CsA and RFP remains to be further elucidated. In addition to P-gp, the drug efflux protein MRP-2 is also a major factor in drug interactions^58^. The upregulation of MRP-2 mRNA expression suggested enhanced drug clearance produced by RFP in both the liver and the proximal tubule equivalents^59^. The cholestasis caused by CsA resulted from the inhibition of hepatobiliary secretion^46^, which was replicated on the cocultured liver spheroids by CsA-induced inhibition of BSEP in a dose-dependent manner. Additionally, the hepatic enzyme AST and the non-organ specific cytotoxic enzyme ALP were decreased by RFP. Nevertheless, TBiL, a serum biomarker of severe liver injury in both human subjects and animals^60,61^, was significantly elevated, although RFP even slightly attenuated the inhibition of BSEP in the present experiment. These results may be due to the test bias in the biochemical analysis methods. Our findings also suggest that we need more specific, accurate and sensitive biomarkers for integrative diagnosis.

The measurement of serum CRE and UREA *in vivo* has long been utilized to diagnose CsA-induced nephrotoxicity^62^; however, these biomarkers do not show secretion for a prolonged period and are thus not sensitive and sufficient, as observed in the present study. The human RPTEC/TERT1 cell line was demonstrated to be capable of polarizing to tight monolayers and expressing tissue-specific enzymes and transporters for a prolonged period and has thus been established as an *in vitro* model for drug safety assessment^9,10,63,64^. The present chip-based liver-proximal tubule coculture model replicated long-term physiological and molecular communication between the two organs and compartments, respectively. The cocultured proximal tubule barrier equivalents in chips expressed several biomarkers, such as KIM-1, CLU, OA, and NGAL, for toxicity assessment over a long period of time, mimicking the *in vivo* responses to nephrotoxic exposures. The FDA and EMEA have approved eight noninvasive renal injury biomarkers, which have been well described *in vivo* and are detectable in urine or blood in animals or humans^21,65,66^. Among the eight qualified biomarkers, KIM-1, albumin, Clu and TFF-3 were considered to be acute tubular alteration-related markers^21^.

In agreement with previous studies^67–70^, the remaining markers that responded to acute CsA-induced nephrotoxicity, including GST-α, NGAL, OPN, GGT, NAG and TIMP-1 in excretory media as well as CRE and UREA in surrogate blood, could be classified as biomarkers for the injury of renal tubular function. OA has been shown to be upregulated in association with acute renal tubule epithelium injury, including CsA-induced injury and tubulointerstitial fibrosis *in vivo*^71^. A dose-dependent increase in OA supported the conclusion that OA can be categorized as a sensitive biomarker for early proximal tubular damage. Urinary α1-MG, which is produced mainly in the liver, can be elevated in both tubular and glomerular injury^72^. Liver-specific FABP (i.e. FABP-1) is expressed in the proximal tubule^73^. These molecules are both biomarkers of acute and especially chronic renal tubular injury, thus, these findings could explain, in part, the elevated FABP-1 and α1-MG, which could only be observed at the late stage of treatment period. Col IV was upregulated by high-dose CsA throughout the study period. Additionally, Col IV is expressed in the renal tubular basement membrane^74^; therefore, the upregulation of Col IV in the coadministration group suggested the regeneration of renal epithelial cells in support of the induction of Ki67 gene expression on day14. CALB is present in the small intestine and will be inhibited in the distal nephron following chronic treatment with CsA^75^. Thus, the response of CALB, a biomarker of renal tubular injury *in vitro* and *in vivo*, to single and repeated treatment with CsA was non-drug-related in the liver-proximal tubule coculture chip. This is the first report of the evaluation of a series of qualified and potential noninvasive nephrotoxicity biomarkers on a microfluidic MOC platform, displaying the capabilities and advantages of MOC in high-throughput screening in early drug development.

The intestine barriers and glomeruli equivalents will be integrated into the novel MOC system, for instance, the four-organ-chip, to improve and optimize our study^11^. On the one hand, the low bioavailability of CsA needs to be taken into account. The low bioavailability of CsA is principally because of the liver metabolism and partly because of the absorption in the intestine. The decreased bioavailability most likely can be explained by an induction of intestinal CYP450 enzymes, which appears to be markedly greater than the induction of hepatic metabolism^20^. The effects of CsA on enterohepatic circulation *in vivo* will be mimicked on the MOC platform.

## Conclusions

The microfluidic multi-organ chip platform assessed in the present study enabled the non-contact cocultivation of reproducible and well-defined liver spheroids and renal proximal tubule barriers, each structure being 1/100,000-fold smaller than their human counterpart organs, in a connecting media circuit over 16 days. Furthermore, the microfluidic device and the designed fluid-to-tissue ratio in the 2-OC model support sufficient inter-organ communication. Finally, the cocultured tissue equivalents expressed tissue-specific markers, enzymes and transporters and could be challenged by CsA with hepatotoxicity and nephrotoxicity which could be ameliorated by the hepatic drug-metabolizing inducer RFP. The present microfluidic chip-based platform fits standardized sizes of microtissues and formats of tissue culture, which can be widely applied in drug screening and is being increasingly accepted by regulatory agencies.

## Materials and methods

### Design and fabrication of the microphysiological 2-OC

The commercially available microphysiological 2-OC^24,33,76^ was integrated by two 96-well cell culture inserts. Fabrication of the 2-OC was performed by TissUse GmbH as described by Wagner *et al*.^24^. Fluid flow in the circuit was established by an on-chip pulsatile micropump modified after Wu *et al*.^77^. The on-chip micropump was actuated by pressured air through an external control unit provided by TissUse GmbH.

### Human cell sources

The human HepaRG cell line and the human proximal tubule cell line RPTEC/TERT1 (CRL-4031) were purchased from ATCC (ATCC, Manassas, VA, USA). HHSteC cell line was purchased from ScienCell Research Laboratories (ScienCell, Carlsbad, CA, USA).

### *De novo* formation of liver equivalents

HepaRG cells were cultured in HepaRG medium (William’s medium E supplemented with 10% fetal bovine serum (FBS, Life Technologies; Sydney, NSW, Australia), 2 mM GlutaMAX, 10,000 U/ml penicillin, 10,000 μg/ml streptomycin, 5 µg/ml human insulin (Sigma-Aldrich, St. Louis, USA) and 5×10–5 M hydrocortisone hemisuccinate (National Institutes for Food and Drug Control, Beijing, China)). Cell culture medium and supplements were purchased from Life Technologies (Life Technologies; Carlsbad, CA, USA). HepaRG cell cultures were at 37 °C and 5% CO_2_, with medium exchange every other day. Differentiation of HepaRG cells was induced by maintaining the cells in the HepaRG medium for two weeks to reach full confluence. Differentiation medium containing 2% dimethyl sulfoxide (DMSO; Sigma-Aldrich; St. Louis, MO, USA) was then added for another two weeks. HHSteC cells were expanded in Stellate Cell Media purchased from ScienCell Research Laboratories (ScienCell, Carlsbad, CA, USA). Cells were harvested for further coculture when they reached 80% confluence.

The formation of human liver spheroids was performed as previously described by Wagner *et al*.^78^. Briefly, 50 μl of cell suspension containing 0.1 × 104 HHSteC cells and 2.4 × 104 HepaRG cells was placed into each well of the 384-well spheroid plate (Corning Costar, Kennebunk, USA). The cells were cultivated in a CO2 incubator on a shaker for 3 days to form round and compact spheroids. The spheroids were then transferred to ultra-low attachment 24-well plates (Corning Costar, Kennebunk, USA). Forty spheroids were collected to form a single liver equivalent for one circuit of the 2-OC.The morphology of differentiated HepaRG cells and the formation of liver spheroids were assessed on an EVOS XL Core digital inverted microscope (Life Technologies, Carlsbad, CA).

### Formation of the renal proximal tubule barrier model

RPTEC/TERT1 cells were cultured in DMEM and Ham’s F-12 (DMEM/F12) medium supplemented with 2 mM GlutaMAX, 10,000 U/ml penicillin, 10,000 μg/ml streptomycin, 36 ng/ml hydrocortisone, 5 μg/ml human insulin, 5 μg/ml transferrin, 5 ng/ml sodium selenite (ITS 1 ×), and 10 ng/ml human epithelial growth factor (hEGF). Medium and supplements were purchased from Life Technologies (Life Technologies; Carlsbad, CA, USA). Hydrocortisone was purchased from Sigma-Aldrich (Sigma-Aldrich, St. Louis, USA).

100, 000 RPTEC/TERT1 cells were seeded on each Transwell permeable support and allowed to attach overnight. Then, medium was exchanged in the top compartment to remove non-adherent cells. Prior to the experiment the seeded supports were cultured for 4 days under static and 3 days under perfused conditions in the 2-OC. Inside the 2-OC the insert membranes were positioned at 100 μm above the bottom of the culture compartment to ensure free media passage below the proximal tubule barriers. Cell morphology and differentiation of RPTEC/TERT1 were monitored using an EVOS XL Core digital inverted microscope (Life Technologies, Carlsbad, CA).

### Chip-based cocultures

Fourteen 2-OC circuits were loaded, and cocultures were cultured in 500 μl circulating medium, and 100 μl medium on top of the barrier insert. The medium in the excretory pool was exchanged daily. Half of the circulating supernatant was replaced and collected. At the end of the 16-day coculture organ-specific functional markers of the liver or proximal tubule equivalent were analyzed by immunofluorescence and qRT-PCR. The on-chip micropump was set to a frequency of 0.8 Hz, resulting in a flow rate that did not exceed 9 μl/min.

### Model compounds

CsA (CAS: 59865-13-3) was added to challenge the cocultures at repeated dose regimens. RFP (CAS: 13292-46-1) administered concomitantly with CsA. For stock solutions, CsA and RFP (National Institutes for Food and Drug Control, Beijing, China) were dissolved in DMSO, stored in the dark at 4 °C, and then diluted to a final concentration of 0.1% DMSO when used.

### Systemic substance exposure to liver-kidney coculture chips

After a three-day administration-free coculture period the CsA stock solution was diluted in fresh medium to the respective concentrations of 10 µM (Low dose) and 20 µM (High dose) and administered daily for 13 days. In the coadministration group 20 µM CsA were applied daily until day6, then 20 µM CsA and 25 µM RFP were administered daily until day13. Medium containing 0.1% DMSO was used for control culture (Fig. 1C).

### Coculture analysis

#### Daily analysis of the supernatant sample

Cell viability was monitored by daily measurement of lactate dehydrogenase (LDH) activity in the supernatants of the liver and proximal tubule barrier compartments. The metabolic activity was detected daily by the measurement of glucose and lactate levels in the supernatants of the two compartments using the Lactate Colorimetric Assay Kit (Biovision, Milpitas, CA, USA) according to the manufacturer’s instructions. On days 1, 7 and 14, LC-MS/MS analysis of the concentrations of CsA and RFP in the surrogate blood circuit and detection of toxicity biomarkers in the supernatants of the two compartments were performed.

The detection of biomarkers, including LDH, glucose, aspartate aminotransferase (AST), alkaline phosphatase (ALP), gamma-glutamyl transpeptidase (GGT), N-acetyl-β-D-glucosaminidase (NAG), total bilirubin (TBiL), albumin (ALB, serum), creatine (CRE) and urea (UREA), was conducted using a 7180 automatic biochemistry analyzer (Hitachi, Japan). The kits for the markers mentioned above were purchased from Wako (Japan). Osteoactivin (OA), glutathione-S-transferase α (GST-α), KIM-1, fatty acid binding protein-1 (FABP-1), collagen IV (Col IV), neutrophil gelatinase-associated lipocalin (NGAL), clusterin (Clu), cystatin C (Cys C), Renin (REN), α1-microglobulin (α1-MG), osteopontin (OPN), neutrophil gelatinase-associated lipocalin (NGAL), calbindin (CALB), tissue inhibitors of metalloproteinases-1 (TIMP-1), interferon-induced protein-10 (IP-10), trefoil factor-3 (TFF-3), epidermal growth factor (EGF) and albumin (urinary) were determined using the MILLIPLEX MAP Human Kidney Injury Magnetic Bead Panel 1 and 2 (Merck Millipore, Billerica, MA, USA). According to the manufacturer’s instructions, the assays were performed on Luminex 200 (Austin, TX, USA. The data were analyzed with MILLIPLEX Analyst 5.1 Software (Billerica, MA, USA).

#### Tissue culture analysis before and after administration

Liver spheroids and membranes holding the RPTEC/TERT1 cells were frozen in O.C.T. Compound and stored at -80 °C until further analysis. For staining sections were fixed with acetone at -20 °C for 10 min or 4% PFA for 10 min. Then, the cells were permeabilized with 0.05% Triton X-100, washed and blocked with 10% goat serum for 30 min., and then incubated with the primary antibody directed against CYP3A4, BSEP, CK8/18, MRP-2, Na^+^-K^+^-ATPase, P-gp, VIM, ZO-1, CLDN 10 and Ki67 (all Abcam, Cambridge, MA, USA) or acetylated-tubulin (Sigma-Aldrich, St. Louis, MO, USA) at 4°Covernight or for 2 h at RT. Thereafter, the sections were washed three times, followed by incubation with the fluorescently labelled secondary antibody at RT for 1 hour. Nuclei were counterstained with DAPI (Sigma-Aldrich, St. Louis, MO, USA).

Proliferation and apoptosis were analyzed by immunofluorescence staining of TUNEL/Ki67. In brief, sections were stained using the TUNEL technique (ApopTag1 Peroxidase *In Situ* Apoptosis Detection Kit, Merck Millipore, Darmstadt, Germany) according to the manufacturer’s instructions. Nuclei were counterstained with DAPI.

All immunofluorescence images were obtained using a Nikon Eclipse 80i Digital Fluorescence Microscope (Tokyo, Japan) and an Olympus IX 71 Inverted Microscope (Tokyo, Japan) with Image-Pro Plus software (version 6.0, Media Cybernetics Inc., MD, USA).

The total RNA in liver spheroids and proximal tubule cells was isolated using the RNeasy^@^ Micro Kit (Qiagen, Duesseldorf, Germany). cDNA synthesis was performed using the FastQuant RT Kit (with gDNase) (Tiangen Biotech; Beijing, China). The qRT-PCR amplifications were performed on Line Gene 9620 model FQD-96A (Bioer) using SYBR Green detection (Tiangen Biotech; Beijing, China). Procedures were carried out according to the manufacturer’s instructions of the kits with the software included in the device. The data analysis was performed with software version 2.0.6. The fold changes in mRNA levels were determined by the 2^-ΔΔCT^ method. The primers were purchased from Sangon Biotech (Beijing, China). TBP and β-actin were housekeeping genes for liver spheroids and proximal tubule barriers, respectively. The primer sequences of target genes are shown in Supple. Tab. 1.

### Statistical analysis

Statistical evaluation was performed using GraphPad Prism (version 6.0, San Diego, CA, USA) and SPSS (version 17.0; IBM, Armonk, NY, USA). *P*-values were calculated by one-way ANOVA followed by Student’s *t* test *post hoc* test. All experiments were performed in triplicate (control and low dose groups) or quadruplicate (high dose and coadministration groups). Unless otherwise indicated, all data are presented as the mean ± SEM.

## Supplementary information

Supplementary Information.
