# Author Correction: Repeated dose multi-drug testing using a microfluidic chip-based coculture of human liver and kidney proximal tubules equivalents

**DOI:** 10.1038/s41598-020-74148-z

**Published:** 2020-10-07

**Authors:** Ni Lin, Xiaobing Zhou, Xingchao Geng, Christopher Drewell, Juliane Hübner, Zuogang Li, Yingli Zhang, Ming Xue, Uwe Marx, Bo Li

**Affiliations:** 1 Key Laboratory of Beijing for Safety Evaluation of Drugs, National Center for Safety Evaluation of Drugs, National Institutes for Food and Drug Control, A8 Hongda Middle Street, Beijing Economic-Technological Development Area, Beijing, 100176 P. R. China; 2 Department of Pharmacology, Beijing Laboratory for Biomedical Detection Technology and Instrument, School of Basic Medical Sciences, Capital Medical University, Beijing, 100069 China; 3 Technische Universitaet Berlin, Institute of Biotechnology, Department Medical Biotechnology, Gustav-Meyer-Allee 25, 13355 Berlin, Germany; 4 TissUse GmbH, Oudenarder Strasse 16, 13347 Berlin, Germany; 5 National Institutes for Food and Drug Control, 31 HuaTuoroad, Daxing district, Beijing, 102629 China; 6 Beijing Institute for Drug Control, 25 Science Park Road, Changping District, Beijing, 102206 China

Correction to: *Scientific Reports*, 10.1038/s41598-020-65817-0, published online 01 June 2020

The original version of this Article contained errors. Xiaobing Zhou was incorrectly affiliated with ‘Beijing Institute for Drug Control, 25 Science Park Road, Changping District, Beijing, 102206, China’. The correct affiliation is listed below:

Key Laboratory of Beijing for Safety Evaluation of Drugs, National Center for Safety Evaluation of Drugs, National Institutes for Food and Drug Control, A8 Hongda Middle Street, Beijing Economic-Technological Development Area, Beijing, 100176, P. R. China

In addition, the Supplementary Information originally published with this Article contained a typographical error in the spelling of the author Juliane Hübner, which was incorrectly given as Juliane Huebner. The name was correct in the original Article.

Finally, the version of this Article previously published contained lower resolution images for Figures 1, 2, 3, 4, 5, 6, 7, 8 and Supplementary Figure 1.

These errors have now been corrected in the PDF and HTML versions of the Article and in the accompanying Supplementary Information file.

