# Supplementary material for: Repeated dose multi-drug testing using a microfluidic chip-based coculture of human liver and kidney proximal tubules equivalents
Source: Sci Rep. 2020 Jun 1;10:8879. doi: 10.1038/s41598-020-65817-0 (PMC7264205; doi:10.1038/s41598-020-65817-0)
Supplement: Supplementary file 1 — Supplementary Information. [file 41598_2020_65817_MOESM1_ESM.docx]

**Supplementary information**

**Repeated dose multi-drug testing using a microfluidic chip-based coculture of human liver and kidney proximal tubules equivalents**

Ni Lin^1, 2‡^, Xiaobing Zhou^1‡^, Xingchao Geng^1^, Christopher Drewell^3^, Juliane Huebner^3^, Zuogang Li^1^, Yingli Zhang^1^, Ming Xue^2*^, Uwe Marx^4*^ and Bo Li^5*^

1. National Center for Safety Evaluation of Drugs, National Institutes for Food and Drug Control, A8 Hongda Middle Street, Beijing Economic-Technological Development Area, Beijing, 100176, P. R. China.
2. Department of Pharmacology, Beijing Laboratory for Biomedical Detection Technology and Instrument, School of Basic Medical Sciences, Capital Medical University, Beijing 100069, China.
3. Technische Universitaet Berlin, Institute of Biotechnology, Department Medical Biotechnology, Gustav-Meyer-Allee 25, 13355 Berlin, Germany.
4. TissUse GmbH, Oudenarder Strasse 16, 13347 Berlin Germany.
5. National Institutes for Food and Drug Control, 31 Hua Tuo road, Daxing district, Beijing 102629, China.

^‡^ N.L., and X.B.Z. contributed equally.

^*^ Corresponding authors:

Bo Li, E-mail: libo@nifdc.org.cn Tel: 010-67095790 Fax: 010-67095790;

Uwe Marx, E-mail: uwe.marx@tissuse.com Tel: 0049-30-5130264-00 Fax: 0049-30-5130264-01;

Ming Xue, E-mail: xuem@ccmu.edu.cn Tel:010-83911520 Fax: 010-83911520.

**Supplementary Figure 1.** Morphology, differentiation of HepaRG cells, and formation of liver spheroids. (A) The morphology of differentiated HepaRG cells. (B) The liver spheroids newly formed and integrated into the compartment of the chip. Scale bar: 100 μm. Characterization of liver spheroids. (C) CK8/18 (green) (D) VIM (green) (E) MRP-2 (red) (F) P-gp (red) (G) BSEP (red) (H Na+-K+-ATPase (red) (I) CYP3A4 (red) (J) ZO-1 (red) (K) TUNEL/Ki67 (green/red) in newly formed spheroids (L) TUNEL/Ki67 (green/red) in spheroids at study endpoint. Nuclei were stained with DAPI (blue). Scale bars A, B, C: 100 μm; D, E, J, and K: 100 μm; F, G, H, I, L and M: 50 μm. qRT-PCR analysis of (M) BSEP (N) Na+-K+-ATPase (O) MRP-2 (P) P-gp (Q) CYP3A4 (R) VIM (S) CK18 (T) Ki67 gene expression. The fold changes in mRNA expression of the liver spheroids at the endpoint are represented relative to the spheroids newly formed; data are the mean± SEM, experiments were performed in triplicates.


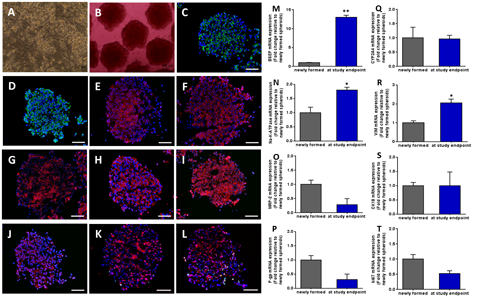


HepaRG cells proliferated, reached confluence and were reproducibly differentiated toward hepatocyte-like and biliary-like cells (Supple. Fig. 1A), as described by Cerec *et al.*[^1^](#_ENREF_1). Immunofluorescence staining of newly formed spheroids (Supple. Fig. 1B) prior to loading into chips was performed. Staining for CK8/18 and VIM demonstrated that HHSteCs were homogenously distributed throughout the spheroid (Supple. Fig. 1C-D). Strong MRP-2, P-gp, BSEP and Na^+^-K^+^-ATPase staining surrounding the cells indicated the polarization of cells and that the liver spheroids completely reformed bile canalicular-like structures (Supple. Fig. 1E-H). Intense staining for CYP3A4 suggested a highly differentiated phenotype and hepatic function (Supple. Fig. 1I). Moderate staining of liver spheroids for ZO-1 demonstrated the expression of intercellular hepatic tight junction-associated proteins (Supple. Fig. 1J). Double-staining for TUNEL/Ki67 revealed that proliferating cells were evenly distributed in newly formed spheroids as well as in those after 16-day 2-OC cocultivation (Supple. Fig. 1K-L), and suggested that the control liver spheroids were not altering dramatically over the course of the experiment.

RT-qPCR analysis of functional hepatic enzymes and transporters BSEP, Na^+^-K^+^-ATPase, MRP-2, P-gp, CYP3A4, VIM, CK18, Ki67 and p53 genes were showed in Supple. Fig. 1M-T, as control of changes in gene expression during a 16-day cultivation in 2-OCs. BSEP showed an up to thirteenfold increase in spheroids after a 16-day cultivation in 2-OCs.The expression of Na^+^-K^+^-ATPase was upregulated by 1.8-fold until day 14, while the expression of MRP-2 and P-gp decreased. The expression of CYP3A4 was maintained during the period of cultivation in the chips. The VIM expression displayed a twofold increase during the 16-day cultivation in the chips, while CK18 maintained the same level of gene expression, probably due to the continuous proliferation of HHSteC cells. In accordance with the change in Ki67 gene expression in control spheroids, p53 gene expression was downregulated by half by the end of the cultivation in the chips, providing evidence that the liver spheroids achieved and maintained a stable differentiated phenotype.

**Supplementary Table 1.** RT-qPCR primers to evaluate gene transcription at the mRNA level after chip culture for liver and proximal tubule equivalents.

| Gene | Primer sequences (5'-3') | |
| --- | --- | --- |
|  | Forward | Reverse |
| CYP3A4 | CTTCATCCAATGGACTGCATAAAT | TCCCAAGTATAACACTCTACACAGACAA |
| PgP/ABCB1 | AAAGCGACTGAATGTTCAGTGG | AATAGATGCCTTTCTGTGCCAG |
| MRP2/ABCC2 | CCCTGCTGTTCGATATACCAATC | TCGAGAGAATCCAGAATAGGGAC |
| BSEP/ACBC11 | AAAAGCACTCATTTGCCCCTG | GTTTCTAGGCTCCCCCAACT |
| Ki67 | GCCCCAACCAAAAGAAAGTCT | AGCTTTGTGCCTTCACTTCCA |
| P53 | TGCGTGTGGAGTATTTGGATG | TGGTACAGTCAGAGCCAACCTC |
| TBP | CCACTCACAGACTCTCACAAC | CTGCGGTACAATCCCAGAACT |
| β-actin | CAAGATCATTGCTCCTCCTGA | AGTCCGCCTAGAAGCATTTG |

**Supplementary Table 2.** List of acronyms.

| Acronym | Definition |
| --- | --- |
| 2D | two dimensional |
| 2-OC | two-organ-chip |
| 3D | [three dimensional](http://www.baidu.com/link?url=rHtGFL_f3J7s6W6OsYpfVrnUaDqi_q9CJ15ms0DiSuYu-vi0EEt_mUXa9gGyw7ePnT3u-nsbDPeuxJouB6Px3q) |
| 3R | replacement, reduction and refinement |
| ALB | albumin |
| ALP | alkaline phosphatase |
| AST | aspartate aminotransferase |
| BSEP | [bile salt export pump](http://www.baidu.com/link?url=I2FkdiC5ON0HXI-KBn9ZixFTsgJF7v3uclsVsI9ChqIZBaI01D-FalJiOczXvsjrytbRpgeeBA9sU0UUpZMCA_) |
| CALB | calbindin |
| CK8/18 | cytokeratin 8/18 |
| Clu | clusterin |
| Col IV | collagen IV |
| CRE | creatine |
| CsA | cyclosporine A |
| CYP3A4 | cytochrome P450 3A4 |
| Cys C | cystatin C |
| DAPI | 2-(4-Amidinophenyl)-6-indolecarbamidine dihydrochloride |
| DMEM/F12 | DMEM and Ham’s F-12 |
| DMSO | dimethyl sulfoxide |
| EGF | epidermal growth factor |
| FABP-1 | fatty acid binding protein-1 |
| FBS | fetal bovine serum |
| GGT | gamma-glutamyl transpeptidase |
| GST-α | glutathione-S-transferase α |
| hEGF | human epithelial growth factor |
| IP-10 | interferon-induced protein-10 |
| ITS | human insulin, transferrin, sodium selenite |
| LDH | lactate dehydrogenase |
| MOC | multi-organ-chip |
| MRP-2 | multi-drug resistance relative protein-2 |
| NAG | N-acetyl-β-D-glucosaminidase |
| NGAL | neutrophil gelatinase-associated lipocalin |
| NGAL | neutrophil gelatinase-associated lipocalin |
| OA | osteoactivin |
| OPN | osteopontin |
| P-gp | [p-glycoprotein](http://www.baidu.com/link?url=JkoXimaKCZDV8GLG1rFgTgC4MI1OKpEVhbMP5IlVN7p-yEOA-jXsshOvL1SZjmM_lGNi2IwWcVE3pW77QcofMa) |
| REN | renin |
| RFP | rifampicin |
| RPTEC/TERT1 | hTERT-immortalized renal proximal tubular epithelial cell |
| RT-Qpcr | real time reverse transcription quantitative polymerase chain reaction |
| TBiL | total bilirubin |
| TBP | TATA-binding protein |
| TFF-3 | trefoil factor-3 |
| TIMP-1 | tissue inhibitors of metalloproteinases-1 |
| TUNEL | terminal dexynucleotidyl transferase (TdT)-mediated dUTP nick end labeling |
| VIM | vimentin |
| ZO-1 | zonula occluden-1 |
| α1-MG | α1-microglobulin |

**Supplementary Table 3.** Observations of the changes in levels of biomarkers in surrogate blood and excretory lumen.

|  | Day 1 | | | Day 7 | | | | Day 14 | | | |
| --- | --- | --- | --- | --- | --- | --- | --- | --- | --- | --- | --- |
| Biomarkers in surrogate blood | Control | CsA 5 μM | CsA 20 μM | Control | CsA 5 μM | CsA 20 μM | CsA 20 μM+RFP 25 μM | Control | CsA 5 μM | CsA 20 μM | CsA 20 μM+RFP 25 μM |
| AST | — | ↑ | ↑ | — | ↓ | ↑/— | ↑*/↑ | — | ↓ | ↑*/— | —/↓# |
| ALP | — | ↑ | ↑ | — | ↑ | ↑/— | ↑/↓ | — | ↑* | ↑*/— | ↑/↓# |
| Tbil | — | ↓ | ↓ | — | — | —/— | ↑**/↑## | — | — | —/— | ↑**/↑## |
| Glucose | — | ↓ | ↓ | — | ↓ | ↓*/— | —/↑ | — | ↓* | ↓**/— | ↓/↑# |
| CRE | — | ↑ | ↑* | — | — | ↓/— | ↓/— | — | ↓ | ↓/— | ↓/↑ |
| UREA | — | ↓ | ↑* | — | ↑ | ↑/— | ↑/↓ | — | ↓ | —/— | ↓/↓ |
| ALB | — | ↑ | — | — | — | —/— | ↓/↓ | — | — | —/— | ↓/↓ |
| GGT | — | ↓ | ↓ | — | ↑ | ↑/— | ↑/— | — | — | ↓/— | ↓/↓ |
| Lactate | — | — | ↑ | — | — | —/— | —/— | — | — | —/— | —/— |
|  | Day 1 | | | Day 7 | | | | Day 14 | | | |
| Biomarkers in excretory lumen | Control | CsA 5 μM | CsA 20 μM | Control | CsA 5 μM | CsA 20 μM | CsA 20 μM+RFP 25 μM | Control | CsA 5 μM | CsA 20 μM | CsA 20 μM+RFP 25 μM |
| GGT | — | — | ↑ | — | — | ↓/— | ↑/↑ | — | ↓ | ↓/— | ↑/↑ |
| ALP | — | — | ↑ | — | — | ↑/— | ↑**/↑# | — | — | ↑/— | ↑*/↑# |
| NAG | — | ↑ | ↑ | — | — | ↑/— | ↑/↑ | — | — | ↑/— | ↑/↑ |
| Glucose | — | ↓ | ↓* | — | — | ↓*/— | ↓**/↓ | — | ↓* | ↓*/— | ↓**/↓ |
| Lactate | — | ↓ | ↑* | — | ↑* | ↑*/— | ↑*/— | — | ↑* | ↑*/— | ↑/↓ |
| KIM-1 | — | ↑ | ↑** | — | ↑ | ↑*/— | ↑/↓ | — | ↑ | ↑/— | ↑/↓# |
| Cystatin | — | ↑* | ↑* | — | ↑ | ↑*/— | ↑/↓ | — | ↑ | ↑/— | ↑/↓ |
| Collagen IV | — | ↑ | ↑** | — | — | ↑*/— | ↑*/↑ | — | ↑* | ↑*/— | ↑*/↑# |
| Clusterin | — | ↑* | ↑* | — | ↑* | ↑*/— | ↓/↓## | — | — | ↑**/— | ↑/↓# |
| NGAL | — | ↑ | ↑* | — | — | ↑**/— | ↑/↓## | — | ↑ | ↑**/— | ↑**/— |
| Osteoactivin | — | ↑* | ↑** | — | — | ↑*/— | ↑*/↑ | — | — | ↑**/— | ↑/↓# |
| IP-10 | — | ↑ | ↑ | — | — | ↑/— | ↑/↑ | — | — | ↑*/— | ↑/↓ |
| GST-α | — | ↑* | ↑* | — | — | ↓/— | ↓/— | — | ↓ | —/— | ↑/↑ |
| albumin | — | ↑ | ↑* | — | ↓ | ↑/— | ↓/↓ | — | ↓ | ↓/— | ↓/— |
| FABP-1 | — | ↑ | ↓ | — | — | —/— | ↓/↓ | — | ↓ | ↑**/— | ↑/↓# |
| Renin | — | — | — | — | ↑* | ↑*/— | —/↓ | — | — | ↑*/— | ↑*/↓ |
| α1-microglobulin | — | ↑ | — | — | ↑ | ↑/— | ↓/↓# | — | ↑ | ↑**/— | ↑/↓# |
| Osteopontin | — | ↑** | ↑* | — | ↑ | ↑*/— | ↓/↓## | — | — | —/— | ↓/↓# |
| TIMP-1 | — | ↑ | ↑* | — | ↑ | ↓/— | ↑/↑ | — | ↓ | ↓**/— | ↓**/↑ |
| EGF | — | — | ↓ | — | ↑ | ↓/— | ↓/↓ | — | ↓ | ↓/— | ↓*/↓ |

Observations of the changes in levels of biomarkers in surrogate blood and excretory lumen after daily administration of CsA alone or with concomitant use of RFP in liver-proximal tubule coculture chips for 14 days. Experiments were performed in triplicates (control and low-dose groups) or quadruplicate (high-dose and coadministration groups). * (#) or ** (##) indicates significant difference p<0.05 or p< 0.01, respectively. * or # indicates differences (increase (↑) or decrease (↓)) compared with the corresponding controls (—) or (/) when the coadministration group was compared with the high-dose group(—), respectively.

1 Cerec, V. et al. Transdifferentiation of hepatocyte-like cells from the human hepatoma HepaRG cell line through bipotent progenitor. Hepatology 45, 957-967, doi:10.1002/hep.21536 (2007).
